# Neuroglial Response to High‐Amplitude, Short‐Duration Pressure Transients in Monoculture

**DOI:** 10.1002/cbf.70231

**Published:** 2026-05-12

**Authors:** J. Logan Jenkins, Pratheepa Kumari Rasiah, Jacob Hardenburger, Wilson Adams, Anita Mahadevan‐Jansen, Bryan Millis, E. Duco Jansen

**Affiliations:** ^1^ Department of Biomedical Engineering Vanderbilt University Nashville Tennessee USA; ^2^ Vanderbilt Biophotonics Center Vanderbilt University Nashville Tennessee USA; ^3^ Department of Neurological Surgery Vanderbilt University Medical Center Nashville Tennessee USA

**Keywords:** blast, calcium signaling, neuroglia, traumatic brain injury

## Abstract

Blast‐induced traumatic brain injury (bTBI) was reported in 125,000 U.S. service men and women from 2000 to 2018. With no prophylactic treatments having been granted FDA approval, there is a clear need for further understanding of the impact of blasts on the central nervous system. The biological response of brain cells due to the near‐instantaneous overpressure of blast onset remains unresolved. Laser‐induced pressures isolate high‐amplitude, short‐duration pressure transients, similar to the initial peak of blast pressures. In this study, we uncover the effects of high‐amplitude, short‐duration pressure transients on monocultures of astrocytes, microglia, and neurons through intracellular calcium imaging, cell viability assays, and quantifying intracellular and extracellular immune signaling proteins. The results indicate that while all three cell types follow a similar activation curve for induced intracellular calcium transients, the downstream impact of high‐amplitude, short‐duration pressures on neurons and glia deviate. Neurons are particularly susceptible to non‐reversible damage, while glia activates traditionally neuroprotective pathways rather than neurodegenerative pathways in response to high‐amplitude, short‐duration pressures. This work has important implications for developing countermeasures for bTBI with either the high‐frequency component of a blast wave or the effects of that component being potential targets for prevention.

## Summary

This study investigates the immediate biological response of neuronal and glial cells in monoculture to short‐duration pressure transients, similar to the initial peak of blast pressures liberated by IEDs (Improvised Explosive Devices). Given that each cell type has unique functional characteristics and responds distinctly to mechanical pressure, the immediate first response and the order of operation within a minute of exposure are crucial to track the forthcoming pathology. These findings enhance the understanding of the compounding effects of pressure on monocultures, and these targets can improve injury predictions and therapeutic opportunities.

## 1 Introduction

Traumatic brain injury (TBI) related to explosive blasts was a leading cause of sustained injuries and mortality in Operation Enduring Freedom (OEF), Operation Iraqi Freedom (OIF), and Operation New Dawn (OND) [1, 2]. The US Department of Homeland Security reports that improvised explosive devices (IEDs) remain the terrorist weapon of choice due to their relative ease of construction, availability, and destructive capacity [3]. While TBIs are a concern for a large, diverse group of individuals, especially adolescents and the elderly [4], military personnel are uniquely at‐risk of blast‐induced traumatic brain injury (bTBI), and the severity is highest in the United States and Canada [5]. Blasts from improvised explosive devices (IEDs) and improvised rocket‐assisted mortars (IRAMS) are a particular threat in combat [6]. Symptoms of bTBI can have devastating impacts on the physical, cognitive, and emotional health of patients. According to statistics from the Defense and Veterans Brain Injury Center (DVBIC), over 383,947 people within the Department of Defense (DoD) suffered a TBI between 2001 and 2018, with more than one‐third exposed to blast events [1, 7]. Currently, no drug‐based treatments for TBIs have passed FDA clinical trials [8]. Therefore, bTBIs remain a focus in research endeavors to better understand the mechanisms of damage and develop potential treatments.

Explosion energy from blasts interacts with the body through the propagation of a large overpressure (a positive pressure transient), sometimes referred to as a “shockwave,” although strictly speaking, only pressure transients that travel at supersonic speed can be classified as true shockwaves. The physiological effects due to this overpressure are characterized as primary, secondary, tertiary, and quaternary blast injuries. Primary blast injury is caused solely by the blast wave interacting with the body, while secondary through quaternary blast injuries are due to later effects of blast, such as injury from projectiles or physical displacement of the body. The mechanisms by which the primary blast injury from direct pressure exposure affects the brain at a cellular level remains unclear. There are a number of methods to replicate blast waves in a research setting, including micro explosions, shock tube, ultrasound, and laser recapitulate the shear forces and injury waves from milliseconds to minutes time scale, some studies have corroborated this with as a real‐world comparison, according to the Conventional Weapons Effect Program (ConWEP) [9, 10, 11, 12, 13, 14]. Laser‐based methods have advantages in creating short, high‐amplitude and high‐frequency pressure waves for mechanistic in vitro studies, this controlled setup allows isolation of these biomechanical effects, allowing for precise spatial targeting of samples that are difficult to obtain with longer‐duration, more physiologically complex blast models, and small fiber‐based form factors for implementation with detection methods. Laser‐induced pressure waves can be generated through several mechanisms of action, each with their own characteristic spatio‐temporal pressure transient profiles: stress confined thermoelastic expansion, secondary to the ablative process as cavitation bubble expansion and collapse, laser‐induced breakdown (LIB) initiated plasma formation, or ablative recoil [15]. In the present study, we have used high‐amplitude, short‐duration pulse transients generated by laser‐induced thermoelastic expansion to specifically study the early cellular effects of this aspect of a mechanical insult. We recognize that these pulse durations are much shorter than the conventional explosion‐induced pressure transients that typically show initial positive peak pressures ranging from 690 to 1724 kPa and with a duration of overpressure of 2–6 ms [16]. Using injury metrics based on short‐duration exposures should be interpreted carefully since the injury mechanisms for long‐duration exposures are likely different, and short‐duration blasts involve smaller momentum transfer, as noted by Karin et al. [17]. However, these shorter‐amplitude simulations of the blast wave can be used to understand the cellular response to mechanical injury in the immediate aftermath of the mechanical insult and to advance knowledge of cellular‐level tolerance criteria.

From a tissue perspective, blast‐damaged portions of the brain can undergo inflammation, ischemia, edema, and vasoconstriction [18]. On the cellular level, the response of neurons to blasts has been extensively studied. Known effects of blasts on neurons include diffuse axonal injury, swollen cell bodies, and neurotransmission loss, cell death [11, 14, 19]. The role of glia in bTBIs remains much less clear than their neuronal counterparts. Astrocytes, the main support cells to neurons, respond to TBIs by maintaining neuronal environments via modulating extracellular osmolarity and ion concentrations, buffering neurotransmitters, and calibrating neurovascular coupling [20]. Microglia, the resident immune cell in the brain, respond to TBIs in either a neuroprotective or neurodegenerative manner. An acute activation of the inflammatory response is vital to the repair and recovery of the brain, while a chronic activation can lead to secondary injury and be detrimental [21].

A ubiquitous indicator of cellular activation across neurons, astrocytes, and microglia is intracellular calcium transients, which drive many physiological functions in neurons, astrocytes, and microglia [22]. In neurons, calcium is a main indicator of excitability, neurotransmission, and metabolism [23]. While in astrocytes, calcium transients are linked to synaptic transmission, neurodegeneration, immune response, brain energy metabolism, and inflammation [24]. Lastly in microglia, intracellular calcium transients play a role in state changes, cytokine production and release, and phagocytosis [25]. Intracellular calcium transients can be induced through multiple sites of origination; either extracellular or intracellular. One manner of calcium initiation is through transient receptor potential (TRP) channels, which are one of the main families of calcium channels that allow an influx of extracellular calcium. Several subtypes of TRP channels are known to be heat and/or pressure‐sensitive [26]. Calcium release originating intracellularly may come from intracellular calcium stores include the endoplasmic reticulum, mitochondria, and lysosomes, which are largely regulated by inositol 1,4,5‐triphosphate (IP_3_) receptors [27].

The calcium transients in glia can modulate several different paths, including cytokine production through intracellular protein nuclear factor kappa B (NF‐κB) [22, 28]. Proinflammatory cytokines, such as interleukin (IL) 2α, IL 2β, IL 7, and TNF‐α, can initiate acute and chronic inflammation with potential beneficial and damaging effects within neurological response to danger stimuli [29]. Other intracellular pathway proteins play major roles in the immune response of microglia and astrocytes. Three other molecular pathways that are relevant to this work are CREB, Akt, and STAT3. cAMP response element‐binding protein (CREB) impacts the immune response by inhibiting NF‐κB activation, inducing macrophage survival, and promoting the proliferation, survival, and regulation of T and B lymphocytes [30]. Protein kinase B (Akt) prevents cell injury, inhibits microglia activation pathway and proliferation, and inhibits apoptotic pathway caspase‐4 [31]. The function of Signal Transducer and Activator of Transcription 3 (STAT3) is more complex since its function can be to promote either pro‐inflammatory or anti‐inflammatory responses through microglial polarization [32].

In this study, primary neurons, astrocytes, and microglia are individually subjected to the initial peak of blast‐induced pressure waves through pulsed laser‐induced pressure generation to better understand the direct impact of fast rise overpressures on each cell type at multiple peak positive pressures. Cellular responses are assessed through induced intracellular calcium transients, cell death by the uptake of necrosis and apoptosis markers, and immune activation through changes in released proinflammatory cytokine concentrations and intracellular pathway messenger protein concentrations.

## 2 Methods and Materials

### 2.1 Cell Culture

Primary astrocytes, microglia, and neuron cultures were prepared in accordance with animal use protocols approved by the Vanderbilt University Institute for Animal Care and Use Committee (VU‐IACUC, Protocol M1600084).

### 2.2 Primary Rat Cortical Astrocytes

Primary astrocytes were isolated from day 0 to 3 postnatal Sprague–Dawley rat pups (Envigo/Harlan, Indianapolis, IN, USA). The neural cortices were mechanically dissected and placed in 1 mL of Dulbecco's Modified Eagle Medium (DMEM) supplemented with 5 mM l‐glutamine, 15% (v/v) fetal bovine serum (FBS), and penicillin/streptomycin (100 U/mL and 100 μg/mL, respectively). Tissues were mechanically dissociated and strained through a 40 μm cell strainer (Falcon, BD Bioscience, Bedford, MA). Strained cells in suspension were plated on culture flasks containing the previously described DMEM. Cells were frozen in liquid nitrogen and thawed for experimentation. Cells were maintained in incubation at 37°C with 5% CO_2_ and 95% relative humidity levels for at least 3 weeks prior to experimentation. After 14 days in vitro, medium concentrations of FBS were reduced to 10% (v/v). Cells were used between days 21 and 35 in vitro for experiments upon re‐plating. Primary astrocytes were detached using a 0.025% trypsin/EDTA solution for 5 min at 37°C and 5% CO_2_ to plate cells for live imaging experiments. Cells were reseeded on glass‐bottom 35 mm cell culture dishes (#0 coverglass, 7 mm diameter, Mattek, Waltham, MA, USA) coated with Poly‐D‐lysine (PDL) at a concentration of about 30,000 cells/cm^2^ [6]. Imaging experiments were performed between 48 and 96 h after re‐plating.

### 2.3 Primary Rat Whole Brain Microglia

P2 dissociated rat whole brain microglia were purchased after isolation from Sprague–Dawley rat pups (Transnetyx, Memphis, TN, USA). Microglia were shipped in vials of Hibernate A (Thermo Fisher Scientific, Waltham, MA, USA) on dry ice. Vials of microglia were placed in a 4°C refrigerator before use for up to 48 h. The Transnetyx protocol was used for microglia plating. In brief, vials of microglia were placed in a 30°C water bath for 1 min before centrifuging 2 mL of microglia in Hibernate A at 200 G. The supernatant was discarded, and cells were resuspended in 1 mL NbActiv1 (Transnetyx, Memphis, TN, USA) with 1× Glutamax and penicillin/streptomycin (100 U/mL and 100 μg/mL, respectively). Cells were counted using an image‐based hemocytometer and Trypan blue in a 1:1 ratio. Cells were plated on a glass 35 mm cell culture dish (#0 glass, 7 mm, Mattek, Waltham, MA, USA) coated with PDL at a concentration of about 50,000 cells/cm^2^. Cells were maintained in incubation at 37°C with 5% CO_2_ and 95% relative humidity levels for 3–7 days until experimentation. Every 3 days, fresh NbActiv1 was added to the dishes in a 1:1 ratio with existing media.

### 2.4 Primary Rat Cortical Neurons

E18 dissociated rat cortical neurons were purchased after isolation from Sprague–Dawley rat pups (Transnetyx, Memphis, TN, USA). The neuron plating protocol mirrors the above microglia protocol, expect cells were plated at a concentration of cells were plated at 16,000 cells/cm^2^. Cells were maintained in incubation at 37°C with 5% CO_2_ and 95% relative humidity levels for 12–26 days until experimentation. Every 3–5 days, astrocyte‐incubated NbActiv1 was added to the dishes in a 1:1 ratio with existing media.

### 2.5 Fluorescence Imaging

#### 2.5.1 Imaging Solutions

Fresh standard imaging solution was composed of the following salts in deionized water (in mM): 140 NaCl, 4 KCl, 2 MgCl_2_, 2 CaCl_2_, 10 HEPES, 5 glucose, pH 7.4 with NaOH, and osmolarity adjusted to ~318 mOsm with mannitol. Ca^2+^‐free extracellular solution was formulated with deionized water containing the following salt concentrations (mM): 140 NaCl, 4 KCl, 4 MgCl2, 10 HEPES, 5 glucose, 0.5 EGTA, pH 7.4 with NaOH, and osmolarity adjusted to ~318 mOsm with mannitol. Stock solutions of pharmacological agents were prepared by vendors by solubilizing them in their appropriate diluent solutions for pharmacological studies. Stock aliquots of Ruthenium red (RR, 10 mM) and 2‐Aminoethoxydiphenylborane (2‐APB, 100 mM) were prepared in water and stored at −30°C. Working concentrations of pharmacological agents for imaging experiments were as follows: RR 10 μM, 2‐APB 100 μM, in physiological saline solution.

### 2.6 Calcium Imaging

**Figure 1:**
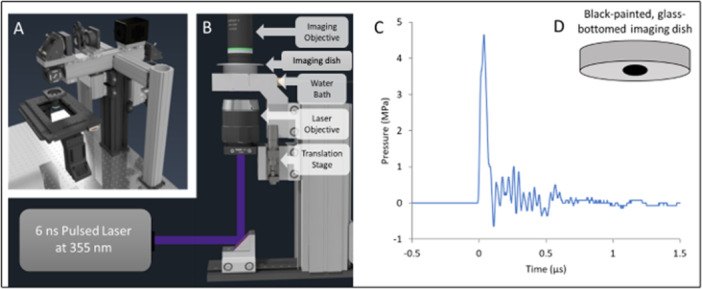
Light and pressure delivery system to a custom‐built widefield microscope. (A) Schematic of the custom‐built upright microscope. (B) Pressure Generation System using a 6 ns pulsed laser (355 nm). (C) Representative pressure transient at the cell surface. (D) Illustration of the 35 mm glass‐bottomed dish.

To image intracellular calcium dynamics of primary rat astrocytes, microglia, and neurons, cells seeded on #0 glass‐bottom imaging dishes were rinsed and incubated with physiological saline solution containing 3 μM of Calbryte 520 AM (AAT Bioquest, Sunnyvale, CA, USA) and for 45–75 min. After dye loading, cells were rinsed with and maintained in a standard bath solution for the duration of imaging sessions (< 15 min). Imaging dishes were placed in a 32°C heated water bath integrated into a custom‐built widefield microscope with 10×, 0.3 NA water‐immersion objective (UMPLFLN, Olympus, Shinjuku City, Tokyo, JP) and ultrafast CMOS camera (ORCA, Hamamatsu, Hamamatsu City, JP) (Figure 1A). Fluorescence excitation was provided by a broadband light source (Lumencore Sola) bandpass filtered to a wavelength range centered around 488 nm. Camera sensor exposure times were held constant at 7 ms for microglia and neuron experiments, and 30 ms for astrocyte experiments.

During an imaging experiment, fluorescence images of dye‐loaded microglia and neurons were acquired at a 2 Hz framerate for 30 s, then 89 Hz frame rate for 0.5 s prior to pressure exposure and 1 s after pressure exposure, and 2 Hz for the last 180 s. Astrocytes were imaged at a 2 Hz framerate for 30 s, then 30 Hz framerate for 0.5 s before pressure exposure and 1 s after pressure exposure, and 2 Hz framerate for the last 180 s. Approximately 30 s into the recording period, cells were exposed to high‐amplitude, short‐duration pressure as described in the Pressure Generation and Measurement methods section. Bath application of pharmacological agents was used to study the influence of molecular signaling pathways involved in high‐amplitude, short‐duration pressure calcium transients. All images were acquired in NIS Elements (Nikon Imaging Systems, Melville, NY, USA) and analyzed using custom processing and analysis workflows with Fiji.

### 2.7 Cell Viability Fluorescence Imaging

Necrosis and apoptosis stains were used to determine the viability of the cell cultures during calcium imaging. Two fluorescent probes were used to better understand the impact of high‐amplitude, short‐duration pressures transients on primary CNS cells in the 35 mm imaging dish. Necrosis stain 1 μM Propidium Iodide (Invitrogen, Waltham, MA, USA) and apoptosis stain 5 μM Biotracker NucView 488 Caspase‐4 dye (Biotium, Fremont, CA, USA) were added to the imaging solution for 10 and 40 min prior to pressure exposure, respectively. For both experiments, a counterstain for nuclei, NucBlue (Invitrogen, Waltham, MA, USA), was used. Fields of views were imaged immediately before laser exposure and 10 and 40 min after exposure to determine signs of cellular necrosis and apoptosis, respectively.

### 2.8 Data Processing

Raw fluorescence traces were extracted using similar methods as [33]. The raw fluorescence traces were temporally aligned via a laser light emission artifact during the pulse, intensity normalized, and processed to extract multiple calcium response metrics. Each cell's raw fluorescence intensity was normalized to the mean raw intensity of the 20 frames preceding pressure exposure, then reported and analyzed as fractions (Δ*F*/*F*). Baseline calcium fluorescence was recorded for at least 30 s prior to pressure exposure. Responses were quantified as Δ*F*/*F* relative to this pre‐injury baseline, with each culture serving as its own control for normalization and statistical comparison. Using the same field of view reduces variability from inter‐cell differences and spontaneous neuronal activity, providing an accurate assessment of pressure‐induced changes in calcium fluorescence Quantitative metrics extracted from each cell's calcium time series trace included maximum change in relative fluorescence (peak Δ*F*/*F*), elapsed time‐to‐peak fluorescence intensity (time‐to‐peak, in seconds), the full‐width half‐maximum of each cell's response (duration, in seconds). A cell was deemed activated if a 10% increase in relative calcium fluorescence within 30 s of pressure exposure was measured. The percentage of activated cells relative to the total number of cells observed is reported as “percentage of responding cells” on a per‐imaging‐experiment basis.

### 2.9 Multiplex Inflammatory Signaling Analysis

The quantification of pro‐inflammatory cytokine concentrations and intracellular pathway protein concentrations were made via multiplex analysis. Pro‐inflammatory cytokine quantification used Milliplex Panel RECYTMAG 75 K (Millipore Sigma, Burlington, MA, USA) and measured by Vanderbilt Hormone Assay & Analytical Services Core using the manufacturer's protocol. Microglia and astrocytes plated on #0 glass‐bottomed 35 mm dishes were exposed to vehicle, 100 ng/mL lipopolysaccharide (LPS), or 4.65 MPa pressure transients. After exposures, cells were aspirated and given just enough volume of media (vehicle for vehicle and pressure groups, LPS media for LPS group) to cover the inner glass well (100 μL). Samples were incubated in normal incubation parameters for 24 h. Dishes of sterile water surrounded the wells during incubation to reduce volume loss due to evaporation. After 24 h of incubation, the entire remaining volume of media (30–50 μL) was collected. Vehicle media was added to the collected samples to bring the volume to 60 μL for each sample. Samples were immediately frozen at −80°C and delivered to the core for analysis. Concentration raw data was normalized to the vehicle group for each of the pro‐inflammatory cytokines.

The quantification of intracellular pathway proteins was made using Milliplex Panel 48 781MAG and measured by Vanderbilt Hormone Assay & Analytical core services using the manufacturer's protocol. Microglia and astrocytes plated in #0 glass‐bottomed 35 mm dishes were exposed to vehicle and 4.65 MPa of high‐amplitude, short‐duration pressure transient. Two and a half hours after exposures, samples were aspirated of all media, washed with ice‐cold PBS once, and 50 μL Milliplex lysis buffer with protease inhibitor (Millipore Sigma, Burlington, MA, USA) was added. Cells were scraped off using the tip of a 50 μL pipette tip and cell suspensions were collected in a microcentrifuge tube and shaken at 4°C for 15 min. Cells were spun at 1000 G using a tabletop centrifuge. Supernatant was collected (~30 μL) and brought to 65 μL with Milliplex lysis buffer. Samples were then frozen at −80°C until transferred to the core. Raw intracellular protein concentrations were normalized by the cell type to the vehicle group for each protein analyzed.

### 2.10 Dye Exclusion Experiments

To examine the effects of injury and Ca^2+^ entry, membrane impermeant, hydrophilic, and fluorescent dye were obtained from Thermo Scientific (10,000 Da TRITC Dextran). Primary rat cortical astrocytes were plated on #0 glass‐bottom imaging dishes, rinsed, and washed with physiological saline solution to remove residual media components. The cells were incubated for 15 min with TRITC Dextran at a final concentration of 1 μg/mL. Immediately after exposure to maximum pressure tested 4.65 MPa (from the previous experiment), cells were flooded with 4% PFA to fix the cells and stop the further cascade of reactions. Cells were fixed for 2 min, and the excess dye was washed thoroughly at least 5 times with 1× PBS. After counterstaining with Hoechst 33342 nucleic acid stain, the cells were imaged to count the percentage of porated cells.

### 2.11 Pressure Generation and Measurement

Pressure transients were generated using a 6 ns pulse from a 355 nm Q‐switched laser (Minilite II, Amplitude Laser Inc., Milpitas, CA, USA). The output of the laser at maximum voltage was 9.0 ± 0.2 mJ per pulse. The 3 mm beam diameter leaving the laser was expanded using a telescope lens system before being brought to the base of the pressure delivery system (Figure 1B). The pressure delivery system consists of an objective on a z‐translation stage, a heated aluminum water bath, and an aluminum sample holder for imaging dish placement. The laser light is delivered to a 20× ReflX objective (Edmund optics, Barrington, NJ, USA) and transmitted through the water bath onto the bottom surface of the glass‐bottomed imaging dish in the sample holder. The laser pulse energy delivered to the imaging dish (after transmission through the various optical elements) was reduced to 1.01 ± 0.07 mJ per pulse mJ/pulse. For pressure generation via ablative recoil, a strong absorber must be present. Black permanent marker (Sharpie PRO Permanent Marker XL, Chisel Tip, Sharpie, Atlanta, GA, USA) was brushed back and forth 5 times on the bottom of glass‐bottom imaging dishes (Figure 1D). The subsequent layer of black absorber was dried for 1 min in a biosafety cabinet prior to incubation. The spot of laser illumination on the black‐painted glass imaging dish was 2 mm in diameter. The black layer strongly absorbed the 355 nm laser light (< 1% transmission). The rapid (6 ns) heating of the absorbing layer due to the absorption of the optical energy, results in a large and fast planar pressure transient that consists of a positive pressure wave of 4.65 MPa maximum peak with a duration of 55 ns (FWHM). Lower pressures were achieved by changing the objective focus location using the z‐translation stage. This changed the laser spotsize and thus the radiant exposure (mJ/mm^2^) delivered to the absorbing layer at the glass surface.

Pressure measurements were taken using a high angle acceptance needle hydrophone with frequency range 1–30 MHz (HNC 300, Onda Corp., Sunnyvale, CA, USA) connected to a hydrophone pre‐amplifier (AH 3010 200, Onda Corp). The voltage signal was transmitted to a Picoscope 3000 series oscilloscope for data collection and converted to pressure in Pa using Onda calibration data. For high‐amplitude, short‐duration pressure calibration, the hydrophone was placed inside a 35 mm imaging dish without cells and brought directly above ( < 1 mm away) the glass of the imaging dish in the same spot used for fluorescence imaging using a 3‐axis micromanipulator. It should be noted that the smaller spikes during the decay phase of the pressure trace following the initial large amplitude spike are likely the result of the hydrophone picking up noise, vibrations of piezo element in the hydrophone due to the primary pressure transient (similar to what is commonly seen when an ideal Friedlander pressure waveform is measured), and/or of acoustic reflections of the primary pressure transients of nearby (tissue) structures that have a different acoustic impedance than the surrounding media. In all cases these additional spikes are nearly an order of magnitude smaller in amplitude than the main positive pressure transient and thus are assumed to not have a significant effect on the cellular responses measured. At least 20 pressure transients for each group were measured leading to a standard deviation of less than 10%.

### 2.12 Statistical Analysis

Statistical comparison of metrics is performed with a two‐sided unpaired Student's *t*‐test. Error bars reported are standard deviation (SD) between imaging experiments. For comparisons involving more than two groups, a one‐way analysis of variance (ANOVA) was performed to determine statistically significant differences among group means. Following a significant ANOVA result, post hoc pairwise comparisons were conducted using the Bonferroni correction. Statistical significance is denoted graphically with asterisks, where * represents *p* < 0.05 and *** represents *p* < 0.001.

## 3 Results

### 3.1 High‐Amplitude, Short‐Duration Pressure Transients Initiate Localized Spots of Increased Intracellular Calcium in Astrocytes

**Figure 2:**
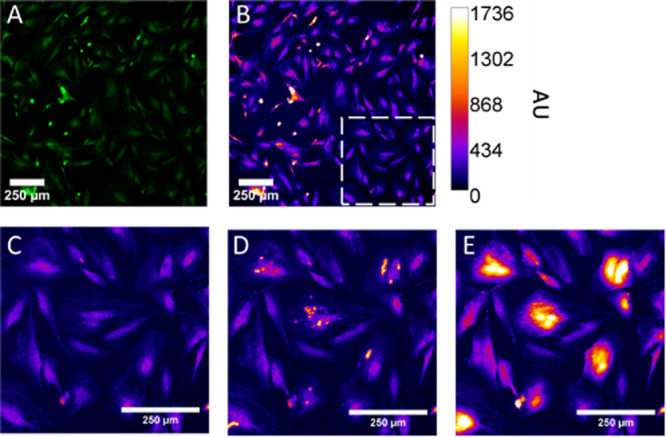
Calcium images of astrocytes in vitro in response to a high‐amplitude, short‐duration pressure transient. (A) Calcium fluorescence prior to exposure is shown in (B), pseudo‐colored to indicate the origin of calcium transients, with intensity represented on the scale in arbitrary units (A.U.). The inset in (B) is enlarged in panels (C–E), showing the temporal progression of calcium signals: pre‐exposure at *t* = −33 ms (C), 33 ms post‐exposure at *t* = 33 ms (D), and 1 s post‐exposure at *t* = 1 s (E).

Figure 2 illustrates the response of astrocytes to a single pressure transient of 4.65 MPa peak positive pressure. The green emitting calcium indicator shows a basal activity in astrocytes in vitro (Figure 2A), though it is more easily seen with false coloring representing the fluorescence intensity (Figure 2B). Using the single fluorescence image taken 33 ms before pressure exposure (Figure 2C) as a comparison, the pressure transient causes localized emissions of fluorescence to appear in the frame (Figure 2D). The calcium fluorescence dissipates to the rest of the cell from these localized spots as seen in the image 1 s after pressure exposure (Figure 2E). Astrocytic calcium responses initiated by these localized spots occurred earlier than calcium responses from adjacent astrocytes without obvious localized calcium increase spots.

**Figure 3:**
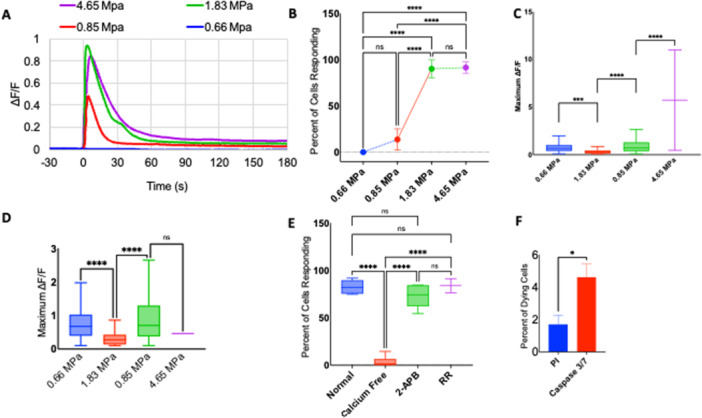
Response of astrocytes in vitro to a laser‐induced pressure wave. (A) Representative calcium responses of astrocytes to four different pressure waves with peak positive pressures of 4.65 MPa (blue), 1.83 MPa (gray), 0.85 MPa (green), and 0.66 MPa (yellow). Calcium responses shown as the change in fluorescence over initial fluorescence (Δ*F*/*F*) from 30 s prior to and 180 s post pressure initiation. (B) Percentage of cells responding (Δ*F*/*F* > 0.1) to four different pressure transients in the entire field of view. (C) Box and whisker plots of maximum Δ*F*/*F* of activated astrocytes in response to three different pressures. (*n* = 400–1000 cells per group). (D) Box and whisker plots of duration of calcium increase as defined by the full‐width, half‐maximum (FWHM) of the calcium transient for three different pressures. (*n* = 400–1000 cells per group). (E) Percentage of cells responding to a 4.65 MPa pressure transient in different media conditions: (1) normal imaging medium, (2) calcium free medium, (3) normal imaging medium with 100 μM 2‐APB, an IP3 receptor inhibitor, and (4) normal imaging medium with 10 μM ruthenium red (RR), a broad TRP channel blocker. (F) Percentage of cells that die after 4.65 MPa pressure over total living cells before as determined by fluorescent necrosis indicator 1 μM propidium iodide and fluorescent apoptosis indicator 5 μM caspase‐3. Data are reported as mean and SD. Student's *t*‐test (***p < 0.001). Box and whisker plots represent the black line as median, x as mean, box lower bound and upper bound as bottom and top quartile, and line bars as minimum and maximum values (excluding outliers). All experiments in the present figure were performed using primary rat cortical astrocytes.

Figure 3 depicts the response of astrocytes to a high‐amplitude, short‐duration pressure transient. Intracellular calcium transients are evoked immediately after exposure to varying peak positive pressures from 0.85 to 4.65 MPa at *t* = 0 s. The intracellular calcium transients are characterized by a sharp rise in Δ*F*/*F* followed by a sharp fall in Δ*F*/*F* to slightly elevated baseline levels of intracellular calcium that lasts for at least several minutes (Figure 3A). The percentage of cells responding to a 0.1 increase in Δ*F*/*F* varies from 0% to 90% of the cells in the fields of view depending on the amplitude of the pressure transient. Fifteen percent of cells in the field of view responded to 0.85 MPa, which increases to the nearly saturated percentage of cells responding to 89% at 1.83 MPa. At 4.65 MPa, 90% of cells respond to high‐amplitude, short‐duration pressure transients well within a variance of the 1.83 MPa pressure transient (Figure 3B). The average maximum Δ*F*/*F* for activated cells increases from 0.85 to 1.83 MPa but. levels off when further increasing the pressure amplitude exposure from 1.83 to 4.65 MPa (Figure 3C). The duration of the calcium response for activated cells is defined as the full width in seconds of half the maximum Δ*F*/*F* (FWHM). The average duration increases for each step in peak positive pressure (Figure 3D). The origin of the calcium response was determined by changing the extracellular environment of the cells. Compared to standard imaging media, calcium‐free media significantly reduces the percentage of cells responding to a 4.65 MPa pressure transient, from 83% to 5%, with a *p*‐value well below 0.001. Imaging media with IP_3_ receptor blocker 100 μM 2‐APB did not significantly affect the number of cells responding to the highest pressure (4.65 MPa) transient. Imaging media with 10 μM RR, a broad TRP channel blocker, also did not significantly change the number of cells responding to 4.65 MPa (Figure 3E). A peak positive pressure of 4.65 MPa increased propidium iodide uptake by only 2% and the uptake of caspase‐4 by less than 5% (Figure 3F).

### 3.2 Microglia Respond to High‐Amplitude, Short‐Duration Pressures via Intracellular Calcium Transients Without Irreversible Damage

**Figure 4:**
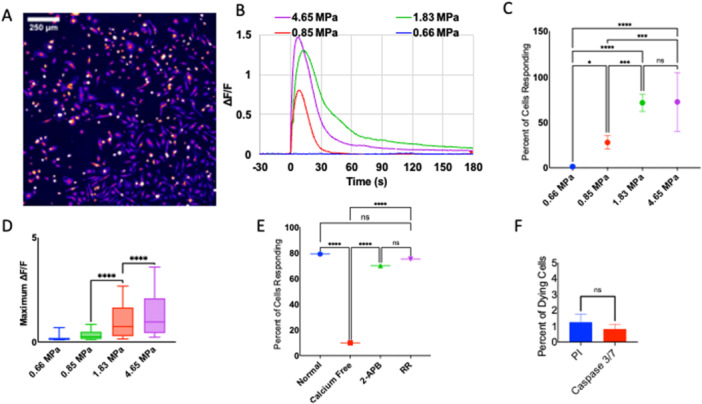
Response of microglia in vitro to a laser‐induced pressure wave. (A) 1.5 mm by 1.5 mm field of view of primary microglia after 4.85 MPa pressure transient with increasing pixel intensity from purple to red to white. (B) Representative calcium responses of microglia to four different pressure waves with peak positive pressures of 4.65 MPa (blue), 1.83 MPa (gray), 0.85 MPa (green), and 0.66 MPa (yellow). Calcium responses shown as the change in fluorescence over initial fluorescence (Δ*F*/*F*) from 30 s prior to and 180 s post pressure initiation. (C) Percentage of cells responding (Δ*F*/*F* > 0.1) to four different pressure transients in the entire field of view. (*n* = 6 dishes per group). (D) Box and whisker plots of maximum Δ*F*/*F* of activated microglia in response to three different pressures. (*n* = 500–1200 cells per group). (E) Percentage of cells responding to a 4.65 MPa pressure transient in different media conditions: (1) normal imaging medium, (2) calcium free medium, (3) normal imaging medium with 100 μM 2‐APB, an IP3 receptor inhibitor, and (4) normal imaging medium with 10 μM ruthenium red (RR), a broad TRP channel blocker. (F) Percentage of cells dying in response to a 4.65 MPa pressure transient as determined by fluorescent necrosis indicator 1 μM propidium iodide and fluorescent apoptosis indicator 5 μM caspase‐3. Data are reported as mean and SD. Student's *t*‐test (***p < 0.001). Box and whisker plots represent the black line as median, x as mean, box lower bound and upper bound as bottom and top quartile, and line bars as minimum and maximum values (excluding outliers). All experiments in the present figure were performed using primary rat whole brain microglia.

Figure 4 illustrates the response of primary microglia to high‐amplitude, short‐duration pressure transients. An image of activated microglia with false coloring representing the fluorescence intensity is shown in Figure 4A. Intracellular calcium transients are evoked immediately after exposure to peak positive pressures from 0.85 to 4.65 MPa. Similar to astrocytes, the calcium response follows a quick rise and fall of Δ*F*/*F* (Figure 4B). The percentage of cells that respond with a greater than 0.1 increase in Δ*F*/*F* varies from 0% to 84% in the fields of view depending on the pressure amplitude. About 14%, 71%, and 85% of cells in the fields of view responded to 0.85, 1.83, and 4.65 MPa peak positive pressure, respectively (Figure 4C). The average maximum Δ*F*/*F* for activated cells increases as the pressure increases (Figure 4D). The origin of the calcium response was determined by modulating the extracellular environment of the cells. In comparison to standard imaging media, calcium‐free media significantly reduces the percentage of cells responding to a 4.65 MPa pressure transient, from 79% to 10%, with a *p*‐value well below 0.001. Imaging media with IP_3_ receptor blocker 2‐APB did not significantly affect the number of cells responding to the highest‐pressure amplitude (4.65 MPa). Imaging media with 10 μM RR similarly did not significantly change the number of cells responding to the 4.65 MPa amplitude pressure transient (Figure 4E). Pressure transients with a peak positive pressure of 4.65 MPa increased propidium iodide uptake by about 1% and the uptake of caspase‐4 by about 1% (Figure 4F).

### 3.3 Neurons Exhibit a Two‐Phenotype Response to High‐Amplitude, Short‐Duration Pressures

**Figure 5:**
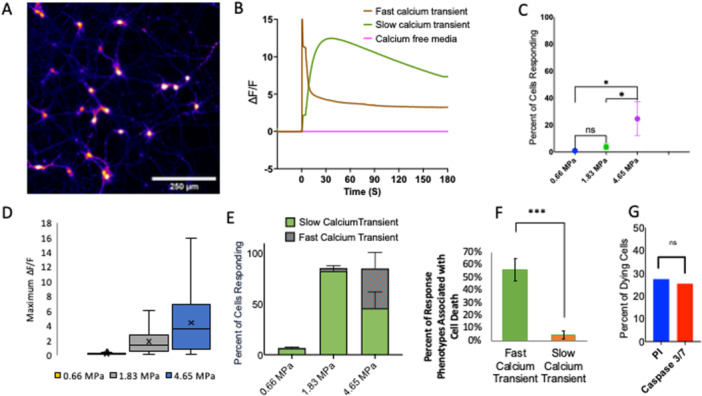
Response of neurons in vitro to a laser‐induced pressure wave. (A) 1.5 mm by 1.5 mm field of view of primary neurons after 4.85 MPa pressure transient with increasing pixel intensity from purple to red to white. (B) Representative calcium responses of neurons to laser‐induced pressure transients demonstrating two phenotypes of response: (1) calcium responses characterized by a fast calcium transient (brown shades), (2) slow calcium transient (green shades), and (3) calcium free media (pink shades). Calcium responses shown as the change in fluorescence over initial fluorescence (Δ*F*/*F*) from 30 s prior to and 180 s post pressure initiation. (C) Percentage of cells responding (Δ*F*/*F* > 0.1) to three different pressure transients in the entire field of view and characterized by calcium response phenotype. Durations less than 180 s (purple) and durations greater than 180 s (solid green). The sum of the two phenotype percentages represents the overall percentage of cells responding. (*n* = 5 dishes per group). (D) Box and whisker plots of maximum Δ*F*/*F* of activated neurons in response to three different pressures. (*n* = < 50 cells for 0.66 MPa, 500–100 cells for higher pressures). (E) Percentage of cells responding (Δ*F*/*F* > 0.1) to three different pressure transients in the entire field of view and characterized by calcium response phenotype. Durations less than 180 s (striped green) and durations greater than 180 s (solid orange). The sum of the two phenotype percentages represents the overall percentage of cells responding. (F) The percentage of each calcium response phenotype associated with subsequent cell death after 4.65 MPa pressure as determined by simultaneous imaging with Calbryte 520 AM and propidium iodide on the same cells. (*n* = 5 dishes). Data are reported as mean and SD. (G) The percentage of cell death after 4.65 MPa pressure was determined by imaging with Caspase 3/7 and propidium iodide in an independent experiment. Data are reported as mean and SD. Box and whisker plots represent the black line as median, x as mean, box lower bound and upper bound as bottom and top quartile, and line bars as minimum and maximum values (excluding outliers). All experiments in the present figure were performed using primary rat cortical neurons.

Figure 5 characterizes the response of primary neurons to high‐amplitude, short‐duration pressure transients. An image of activated neurons with false coloring representing the fluorescence intensity is shown in Figure 5A. Intracellular calcium transients are evoked immediately after exposure to peak positive pressures from 1.83 to 5.65 MPa. Unlike the responses from glia, two phenotypes of response emerged, particularly when exposed to the highest amplitude pressure of 4.65 MPa. One phenotype can be described as a “fast calcium transient” and is characterized by a calcium transient that peaks within 60 s and is reduced to at least half the maximum Δ*F*/*F* by 180 s. The other phenotype is described as a “slow calcium transient” characterized by a calcium transient above 0.1 Δ*F*/*F* and does not reduce below half the Δ*F*/*F* by 180 s. Typical “fast calcium transients” and “slow calcium transients” are shown in Figure 5B. However, in a Ca2+ free imaging solution, Δ*F*/*F* remained negligible. The percentage of cells that respond to high‐amplitude, short‐duration pressure transients increase from < 5% at 0.66 MPa to 80% at 1.83 MPa and the dominating phenotype are the “slow” calcium transients. At 4.65 MPa, the overall percentage of cells responding does not change from 1.83 MPa, but the ratio of fast to slow calcium transient phenotypes does. The ratio of fast to slow calcium transients is nearly 1:1 at 4.65 MPa with the “slow calcium transient” phenotype making up 54% of the cells responding and the “fast calcium transient” consisting of 46% of the cells responding (Figure 5E). As with the glia cells, an increase in peak positive pressure causes an increase in the maximum Δ*F*/*F* (Figure 5D). The cell viability changes significantly from 1.83 MPa, where nearly no cells experience necrosis due to pressure exposure, to 4.65 MPa where about 27% of cells die within 10 min of pressure exposure (Figure 5F). Due to the use of simultaneous calcium and propidium iodide imaging, an association between calcium transient phenotypes and their subsequent cell viability can be made. Most cell death occurred in cells initiating a “fast calcium transient” in response to the 4.65 MPa pressure pulse. Fifty‐five percent of all cells exhibiting a “fast calcium transient” phenotype were necrotic 10 min post‐pressure exposure, while only 4% of cells exhibiting the “slow calcium transient” phenotype took the propidium iodide dye (Figure 5F).

### 3.4 Comparing the Responses of Astrocytes, Microglia, and Neurons to High‐Amplitude, Short‐Duration Blast

**Figure 6:**
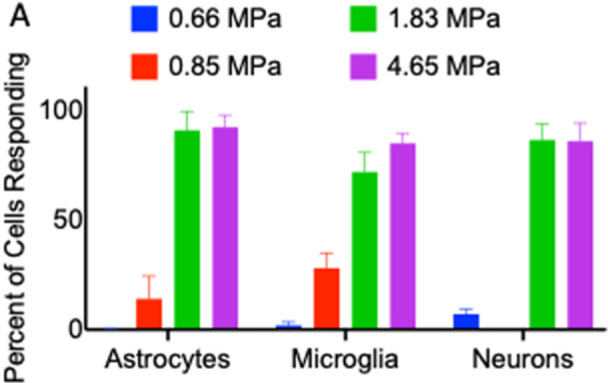
Comparison of responses of primary astrocytes, microglia, and neurons to laser‐induced pressure transients. (A) Percentage of astrocyte (orange), microglia (blue), and neurons (green) responding (Δ*F*/*F* > 0.1) to four different pressure transients at 0.66, 0.85, 1.83, and 4.65 MPa peak positive pressure. Neurons were not subjected to 0.88 MPa pressure transients. Data are reported as mean and SD. Student's *t*‐test (***p < 0.001).

A comparison of the responses of each cell type is provided in Figure 6. All three cell types increase in percentage of cells responding to pressure as the peak positive pressure is increased (Figure 6A). In this study, the term activation is used to describe an initial mechanically induced cellular response to pressure wave exposure, rather than classical inflammatory activation characterized by cytokine expression. Less than 10% of all cell types activate at the lowest pressure of 0.66 MPa and all cell types are near saturated percent‐activated at 1.83 MPa peak positive pressure. Comparing the percentage of cells that die within 10 min of 4.65 MPa pressure exposure using propidium iodide shows a clear difference between neurons and glia. Apoptosis stain caspase‐4 reveals a small but significant difference in the percentage of cells that take up the dye 40 min after pressure exposure between microglia, 1%, and astrocytes, 5%. The mechanism by which intracellular calcium transients are initiated appears largely due to extracellular calcium for astrocytes and microglia, as less than 10% of cells activated in the absence of extracellular calcium.

### 3.5 Immune Responses of Astrocytes and Microglia

**Figure 7:**
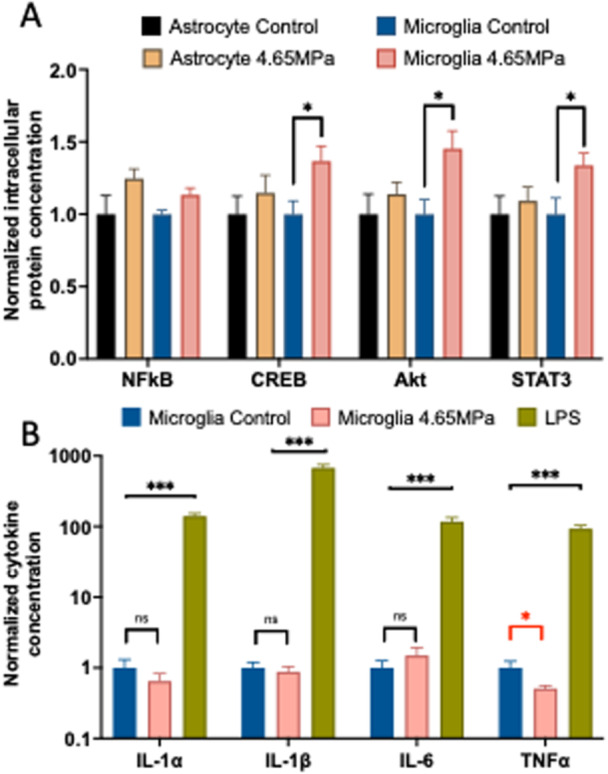
Effects of high‐amplitude, short‐duration pressure transients (4.65 MPa) on intracellular protein concentrations and proinflammatory cytokine release. (A) Total intracellular protein concentrations of CREB, NF‐κB, Akt, and STAT3 collected from astrocyte (solid color) and microglia (striped color) cell cultures 2.5 h after high‐amplitude, short‐duration pressure transients of 4.65 MPa (blue) or sham exposure (0 MPa) (control, red). Intracellular protein concentrations are normalized to each groups' respective control. (B) Proinflammatory cytokine concentrations released from microglia were collected 24 h after sham (red), positive control (100 ng/mL lipopolysaccharide (LPS) exposure (purple)), and 4.65 MPa of high‐amplitude, short‐duration pressure transients (blue). Released cytokine concentrations are normalized to each group's respective negative control. Data are reported as mean and SD. Student's *t*‐test (*p < 0.05, ***p < 0.001). In Figure 7B, the statistical significance denoted in red was performed as an unpaired Student's *t*‐test, *p* < 0.05.

Figure 7 highlights the immunological signaling responses of astrocytes and microglia to 4.65 MPa pressure exposure. Astrocytes produced small but insignificant changes in concentrations of intracellular proteins NF‐κB, CREB, Akt, and STAT3, 180 min after 4.65 MPa pressure exposure. Microglia did not show a significant increase in concentration of proinflammatory mediator NF‐κB due to a 4.65 MPa pressure transient but did have significant (*p* < 0.05) protein concentration increases of 33%, 45%, and 34% in CREB, Akt, and STAT3, respectively, 180 min after 4.65 MPa pressure exposure (Figure 7A). Twenty‐four hours after pressure exposure, concentrations of released proinflammatory cytokines IL‐2a, IL‐2b, TNF‐α, and IL‐7 did not significantly change (Figure 7B) in comparison to a positive control for the pro‐inflammatory response of primary microglia (100 ng/mL lipopolysaccharide (LPS) added to culture media 24 h prior to collection). However, the only cytokine TNF‐α seems significantly reduced after pressure exposure of 4.65 MPa. The concentrations of the four pro‐inflammatory cytokines increased by at least two magnitudes in response to 24 h of LPS exposure.

### 3.6 Rapid Poration on the Cell Membrane Facilitates External Calcium Entry in Astrocytes

**Figure 8:**
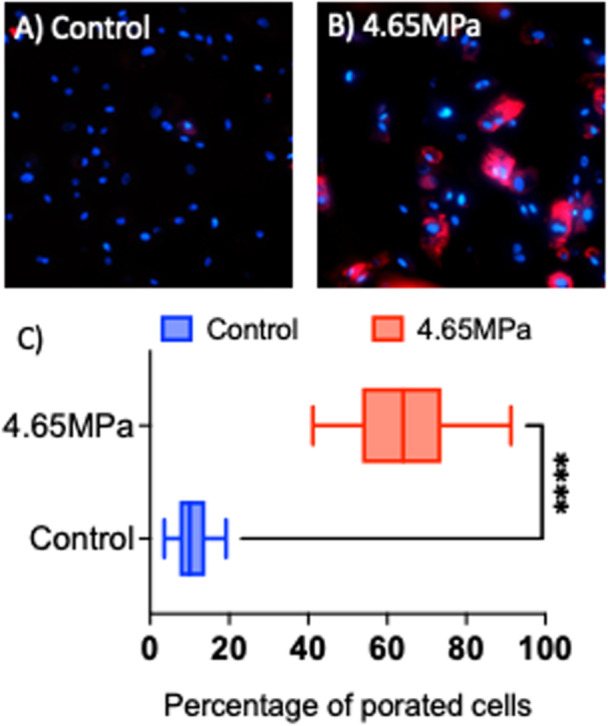
Dye inclusion on astrocytes exposed to a maximum peak pressure (4.65 MPa). (A and B) Representative images of cells counterstained with DAPI (blue), 10 kDa dextran (red), cells were fixed 10 s post‐exposure. (C) Percentage of cells that had dextran permeated into the cytoplasm. *N* = 500 cells per experimental condition. ****p < 0.0001.

Cell membranes are impermeable to dyes; however, Figure 8 shows the differential uptake of TRITC‐Dex by astrocytes after exposure to 4.65 MPa pressure. Control or unexposed cells showed a minimal adherence of the dye to the surface and no cytoplasmic residues were identified after several washes. In contrast, in the pressure exposued cells, approximately 65% cells had an intracellular inclusion of 10 kDa TRITC Dex, clear evidence of rapid poration, disruption of membrane, and intracellular inclusion of the dye.

## 4 Discussion

This work aims to better understand the effects of high‐amplitude, short‐duration pressure transients on different cell types in the brain in the immediate timeframe post‐exposure through intracellular calcium imaging and short‐term immune function assessments. We have demonstrated: (1) a similarity in astrocytes, microglia, and neurons to stimulate intracellular calcium transients to full effect at 1.83 MPa pressure exposure from the described high‐amplitude, short‐duration pressure transients, with nearly no activation at 0.66 MPa pressure exposure, (2) a similarity in astrocytes and microglia calcium transient characteristics in which the increased intracellular calcium quickly rises and falls within 30–60 s, (3) neuronal calcium transients that are characteristically different from the glial calcium transients and in which there are two transient phenotypes of neuronal calcium activation; fast and slow, (4) a similarity in glia cell calcium origination as extracellular calcium is necessary for calcium activation in both microglia and astrocytes, but is not TRP channel‐dependent in either, (5) a similarity in glia cells in which they are resistant to cell necrosis the first 10 min post exposure and apoptosis the first 40 min post exposure up to 4.65 MPa, (6) a difference between glia and neurons, where pressure transients at 4.65 MPa cause irreversible damage in a significant fraction of neurons but not in glia, (7) a significant increase in microglia intracellular pathway proteins CREB, Akt, and STAT3, but a nonsignificant increase in cytokine precursor NF‐κB, and (8) a lack of released proinflammatory cytokine increase of IL‐2α, IL‐2β, IL‐7, and TNF‐α 24 h after pressure introduction in primary microglia.

### 4.1 Astrocytes and Microglia Respond to High‐Amplitude, Short‐Duration Pressure Exposures via Intracellular Calcium Transients

Intracellular calcium transients are a ubiquitous second messenger of many physiological responses, but it's the spatio‐temporal patterning and decoding of the transient that determines downstream implications [25]. The calcium responses of astrocytes and microglia to high‐amplitude, short‐duration pressure exposures share many similarities, including the percentage of cells responding at varying pressures, time to peak Δ*F*/*F*, and duration of responses. The trends of increasing maximum Δ*F*/*F* as pressure increases (until saturation) and increasing duration as pressure increases (until saturation) are present in both glial cells as well. The similarities in spatio‐temporal patterning suggest that a common pathway of pressure‐induced intracellular calcium transients may exist. As glial cells function to support and maintain the health of the CNS, astrocytes and microglia share many functional pathways. The specific nature of that pathway is not clear, and more work is needed to definitively answer this question.

Current literature on blast‐mediated calcium transients suggests purinergic signaling [34] and mechanosensitive channels [35] are the main proponents of calcium activation. Purinergic signaling may not be the target of high‐amplitude, short‐duration pressure transients due to complimentary observations within this work. The extracellular ATP needed to initiate purinergic signaling is thought to be released from the high concentrations of basal ATP in cells during damage and cell death. In these experiments our microglial and astrocyte demonstrated only limited cell death in monocultures [36]. It is possible that increased extracellular ATP occurs through non‐damage mechanisms, but we present no evidence to support this. In general, cell‐to‐cell signaling is unlikely to be a cause of the immediate calcium response since the diffusion speed extracellularly [37] is on the order of 10 μm/s and intracellular calcium transients were observed within 11–43 ms post‐pressure. This would result in signaling molecules moving less than 1 μm in that time, which is shorter than cell‐to‐cell separations in these experiments. A main mechanosensitive family of channels, TRP channels, was determined to have little effect on the ability of astrocytes and microglia to induce intracellular calcium transients after high‐amplitude, short‐duration pressure transients via broad TRP blocker, ruthenium red; meaning that TRP channels cannot be the sole mechanism of extracellular calcium influx, if they are involved at all. There was a small, but significant decrease in the average maximum Δ*F*/*F* in the presence of RR for microglia (data not shown) that may suggest a role of TRP channels in maximizing intracellular calcium responses, but the trend of low TRP involvement for initiation purposes remains.

An unexpected result from this work was the observation of localized, subcellular concentrations of increased intracellular calcium in astrocytes, and to a lesser extent microglia, which manifest themselves as fluorescence “hot spots” within 11–43 ms of pressure exposure (Figure 2). Given the extracellular origin of this response, one theory for this observation is the induction of micropores or nanopores in the bilipid membrane. As the name implies, nanopores are nanometer sized holes. Qualitatively, the localized spots can appear both near and far from nucleus, and the number of pores can range from 1 to 20 per cell. A channel‐based mechanism would likely occur at many locations throughout the cells. A study [37] on nanoelectroporation‐induced calcium transients in mammalian cells demonstrates similar calcium transient characteristics, such as sub‐60 s durations and response onsets within seconds of stimuli, as our experiments demonstrated as well. The fluorescent dye inclusion experiment provides intel on the rapid intracellular entry and retention of 10 kDa Dex molecules. Presumably, the extracellular calcium facilitated by membrane nanoporation could potentially be the first millisecond responders of a bTBI. Further investigation is needed to better understand the meaning of these localized increases in intracellular calcium to determine calcium origin and functional effects.

### 4.2 Neuronal Intracellular Calcium Responses to High‐Amplitude, Short‐Duration Pressure Exposures

Neurons, being the base unit for the entire nervous system, could cause profound, detrimental effects to brain function if affected by high‐amplitude, short‐duration pressures. Unlike astrocytes and microglia, neurons are non‐dividing cells and damage due to blast may be permanent. Unlike the glial intracellular calcium responses at the same pressure exposures, neurons exhibited two phenotypes of calcium transients, especially at 4.65 MPa pressure exposures. The “fast calcium transient” phenotype, while often similar in shape and duration as glial calcium responses, was strongly associated with subsequent cell death via necrosis staining. This is unlike the glial calcium transients as only small changes in cellular viability were observed. The “slow calcium transient” phenotype observed in neurons differs greatly in overall shape and duration from their glial counterparts, suggesting a different physiological pathway may be involved. It is also notable that the two phenotypes may be independent of one another, as the “slow calcium transient” phenotype was observed without many cells presenting the “fast calcium transient phenotype.” If true, the dual responses of neurons may be purely a function of the photomechanical insult rather than cell‐to‐cell communication. The functions of the neuronal “slow calcium transient” phenotype need further investigation, though known functions of calcium transients in neurons can guide the prediction of potential downstream effects. An obvious role of intracellular calcium transients in neurons is neuroexcitability, but calcium transients with durations over 100 s from high‐amplitude, short‐duration pressure differ greatly from normal neuronal spiking of only a few seconds [22, 38]. The origin of high‐amplitude, short‐duration pressure calcium transients in literature remains unclear, with separate studies suggesting extracellular origin [26] and intracellular origin [39].

### 4.3 High‐Amplitude, Short‐Duration Pressure Transients Can Affect Cell Viability

Cell death is a major driver of pathology for bTBI. Blast‐mediated damage can occur in neurons, astrocytes, and microglia, with a particularly damaging effect on neurons [40, 41] (Rama Rao et al., 2018). The effects of high‐amplitude, short‐duration pressure transients on CNS cell health have not been well defined. This work highlights the heightened sensitivity of primary neurons to high‐amplitude, short‐duration pressure exposures as 27% of neurons up took necrosis indicator, propidium iodide, 10 min after 4.65 MPa pressure exposure compared to 2% and 1% for astrocytes and microglia, respectively. Only a small percentage of astrocytes, ~5%, and microglia, 1%, showed signs of apoptosis 40 min after 4.65 MPa pressure exposure [42, 43]. The “fast calcium transient” phenotype of neurons described in this work was highly associated with later necrosis. The short duration of this response may be due to the loss of integrity of the bilipid membrane in which the calcium reporter would no longer be spatially confined. This would result in a decrease in baseline calcium fluorescence after pressure transients, which was observed in a subset of the phenotype's responders.

This work suggests neurodegenerative events, such as astrocyte and microglia reactivity, neuroinflammation, and excitotoxicity [42, 43], may be driven solely by secondary effects from neuronal cell death rather than primary effects of blast on glia. This could be further investigated using co‐cultures of various CNS cell types to better understand their interconnected responses. A limitation to this work is the cell viability ramifications of high‐amplitude, short‐duration pressure exposures were investigated for only 40 min after pressure exposure. While caspase‐4 is a main driver of apoptosis, it is possible that other apoptosis pathways are activated by these pressure characteristics.

### 4.4 Immune Signaling Responses of Microglia and Astrocytes to High‐Amplitude, Short‐Duration Pressure Transients

Ultimately, the debilitating effects of bTBI are long‐term changes in anatomy and physiology, including neuroinflammation and its impact on the brain. The mechanism behind the resultant inflammation has been described as both a direct effect [21] and solely an indirect [44] effect of blast overpressures. Therefore, this work investigated the possible activation of immune response in astrocytes and microglia through the increase of intracellular pathway proteins and the release of proinflammatory cytokines. Microglia showed a significant increase in intracellular concentrations of CREB, Akt, and STAT3 when measured 2.5 h after a 4.65 MPa pressure transient. The significance of these three intracellular proteins is they all can drive microglia into a neuroprotective state: promoting proliferation, suppressing inflammation, clearing dead cells, and aiding neurogenesis and tissue recovery. The impact of STAT3 is more complex as it has pro‐inflammatory and anti‐inflammatory properties [32]. Other modalities to study TBI converge to a similar finding [19, 45, 46], that glial cells initially are neuroprotective after injury, though most findings occurred in vivo [32]. In contrast, the proinflammatory precursor NF‐κB did not increase significantly in microglia after a high‐amplitude, short‐duration pressure transient. Further validation of high‐amplitude, short‐duration pressure transients lack of initial proinflammatory response comes as there were no significant changes in released proinflammatory cytokine concentrations, IL‐2α, IL‐2β, IL‐7, and TNF‐α 24 h after pressure exposure.

Astrocytes also play an important role in the immune response of the central nervous system. That same immune signaling proteins were quantified for astrocytes and increases in neuroprotective pathway proteins were not observed after pressure exposure. It should be noted that other researchers have found increases in CREB, Akt, and STAT3 [47, 48] concentrations in astrocytes after TBI initiation. This discrepancy may be due to the early measurement point of 2.5 h after pressure exposure and further investigation into the temporal aspect of intracellular pathway protein upregulation would yield similar results.

From this work we propose high‐amplitude, short‐duration pressure transients stimulate microglial neuroprotective functions within hours after insult. Most TBI literature uses an in vivo model system to investigate immune and inflammatory responses. However, the study holds a limitation of using monoculture systems to isolate cell type specific responses to defined pressure transients. However, responses in monoculture may differ substantially from those in co‐culture or multicellular systems due to the absence of intercellular signaling. Therefore, the findings should be interpreted as complementary to, rather than fully representative of, multicellular responses. This work suggests that neuroprotective states may be at least partially activated due to direct overpressure of a blast rather than only responses to neuronal damage or other extracellular signals.

### 4.5 Comparing High‐Amplitude, Short‐Duration Pressure to Other Blast Modalities

The high‐amplitude, short‐duration pressure transient in this work is used to mimic the initial peak of pressure due to a blast event. The initial peak of explosive blasts in the field has maximum pressures on the order of 10's to 100's of kPa instead of single MPa and durations on the order of single μs rather than sub 200 ns [49]. The need for higher maximum peak pressures in our work is likely due to multiple mechanisms driving the impact of blast on the brain. The multitude of factors that can impact the brain during blast include head acceleration, deacceleration, impulse, and shear strain [1]. The impulse, that is the integral of pressure over time (P•s), is thought to be a main driver of TBI‐associated damage [50]. The impulse in this work is 1–3 magnitudes lower than impulses generated by shock tube needed to induce bTBI allowing scaling to an in vitro setup [40]. The pressure transients generated in this model are not intended to replicate the full temporal characteristics of field‐scale blast exposures; the use of high‐peak, short‐duration loading is a deliberate experimental choice aimed at isolating specific injury‐relevant mechanisms. In particular, these transients enable controlled application of extreme overpressure at high strain rates while minimizing confounding effects of prolonged impulse and complex wave reflections inherent to large‐scale blast environments. As a result, this approach is not designed for direct clinical translation of blast injury thresholds but rather for mechanistic interrogation of rapid mechanical loading effects at the cellular and subcellular levels. Insights gained from such controlled loading paradigms are essential for informing multiscale injury models and refining tolerance criteria that ultimately support, rather than replace, clinically translational blast studies. The current findings provide mechanistic insights into how high‐amplitude, short‐duration pressures affect neuronal and glial responses, in particular immediately following exposure to pressure transients, while direct clinical translation is limited. Understanding these fundamental responses is a critical step toward designing strategies that could ultimately reduce the severity or frequency of bTBI. The controlled high‐amplitude, short‐duration exposures allow for precise investigation of impulse‐related injury mechanisms that cannot be isolated in more complex physiological models. The insights gained here represent a foundational understanding of early phase cellular responses to biomechanical insults, which is a necessary step toward future studies with clinical relevance.

## 5 Conclusion

In conclusion, we have demonstrated the impact of high‐amplitude, short‐duration pressure transients on monocultures of astrocytes, microglia, and neurons and their individual roles in short‐term physiological responses. Specifically, we found that (1) a similar percentage of cells from all three types respond to each peak positive pressure tested, (2) astrocytes and microglia respond similarly to high‐amplitude, short‐duration exposures in calcium transient characteristics, origin of calcium response, and lack of proinflammatory response, (3) microglia drive neuroprotective pathways in response to these pressure transients, (4) neurons initiate two phenotypes of calcium response, one of which is highly associated with cell damage, and (5) neurons are most susceptible to immediate cell death following high‐amplitude, short‐duration pressure transients once the amplitude exceeds a threshold (1.83 MPa). Through this work, we now understand that the initial peak in pressure transients associated with blast may be capable of directly eliciting physiological responses and damage in CNS cells. The negative effects of primary blast may be most driven by damaged neurons and not the neuroinflammatory impact of glial cells. Evidence from literature indicates that neurons are highly vulnerable cells that are easily damaged, exhibit limited regenerative capacity, and are prone to irreversible cell death following injury [51]. In contrast, glial cells play a critical supportive role by maintaining neuronal homeostasis and providing metabolic, structural, and protective support. Glial cells are comparatively resilient and function as a buffering and cushioning system that preserves neuronal integrity. Among glial populations, astrocytes are recognized as key secondary responders to injury, where they actively participate in regulating the cellular and molecular responses to neural damage [52]. Our monoculture experiments indicate that neurons are significantly more susceptible to injury induced cell death than astrocytes under the conditions tested. Literature evidence supports the notion that high‐amplitude blast waves preferentially impact neurons, while glial cells demonstrate relatively greater resilience. Nevertheless, further investigation using coculture models is warranted to clarify the temporal sequence and interplay of cellular responses. Countermeasures designed to reduce the high frequency component of blast or reduce the neuronal susceptibility to high‐amplitude, short‐duration component of blast may help reduce the severity and number of bTBIs on the battlefield.

## Author Contributions

**J. Logan Jenkins:** investigation, methodology, data curation, writing – original draft preparation. **Pratheepa Kumari Rasiah:** investigation, data curation, review and editing. **Jacob Hardenburger:** methodology, data curation. **Wilson Adams:** methodology, data curation. **Anita Mahadevan‐Jansen:** methodology, validation, conceptualization. **Bryan Millis:** methodology, data curation, validation, conceptualization. **E. Duco Jansen:** funding acquisition, validation, conceptualization, funding acquisition, writing and review.

## Transparency, Rigor, and Reproducibility Statement

In our study, we made sure to provide transparency and rigor by thoroughly outlining all experimental protocols, data collection methods, and statistical analyses in the manuscript. This enables anyone to replicate our work fully. Furthermore, all data and code used in the study will be accessible upon request, facilitating reproducibility and further research in the field.

## Data Availability

The data that support the findings of this study are available from the corresponding author upon reasonable request.

## References

[1] D. W. Bryden , J. I. Tilghman , and S. R. Hinds , “Blast‐Related Traumatic Brain Injury: Current Concepts and Research Considerations,” Journal of Experimental Neuroscience 13 (2019): 1179069519872213, 10.1177/1179069519872213. 31548796 PMC6743194

[2] V. G. Coronado , L. C. McGuire , K. Sarmiento , et al., “Trends in Traumatic Brain Injury in the U.S. and the Public Health Response: 1995–2009,” Journal of Safety Research 43 (2012): 299–307, 10.1016/j.jsr.2012.08.011. 23127680

[3] Improvised Explosive Devices (IEDs)|Homeland Security, (n.d.) , accessed September 2, 2023. https://www.dhs.gov/topic/explosives.

[4] S. R. Flanagan , “Invited Commentary on “Centers for Disease Control and Prevention Report to Congress: Traumatic Brain Injury in the United States: Epidemiology and Rehabilitation,” Archives of Physical Medicine and Rehabilitation 96 (2015): 1753–1755, 10.1016/j.apmr.2015.07.001. 26184889

[5] J. Haarbauer‐Krupa , M. J. Pugh , E. M. Prager , N. Harmon , J. Wolfe , and K. Yaffe , “Epidemiology of Chronic Effects of Traumatic Brain Injury,” Journal of Neurotrauma 38 (2021): 3235–3247, 10.1089/neu.2021.0062. 33947273 PMC9122127

[6] G. Ling , F. Bandak , R. Armonda , G. Grant , and J. Ecklund , “Explosive Blast Neurotrauma,” Journal of Neurotrauma 26 (2009): 815–825, 10.1089/neu.2007.0484. 19397423

[7] G. S. Ling and J. M. Ecklund , “Traumatic Brain Injury in Modern War,” Current Opinion in Anaesthesiology 24 (2011): 124–130, 10.1097/ACO.0b013e32834458da. 21301332

[8] A. I. R. Maas , B. Roozenbeek , and G. T. Manley , “Clinical Trials in Traumatic Brain Injury: Past Experience and Current Developments,” Neurotherapeutics 7 (2010): 115–126, 10.1016/j.nurt.2009.10.022. 20129503 PMC5084118

[9] A. Nakagawa , G. T. Manley , A. D. Gean , et al., “Mechanisms of Primary Blast‐Induced Traumatic Brain Injury: Insights From Shock‐Wave Research,” Journal of Neurotrauma 28 (2011): 1101–1119, 10.1089/neu.2010.1442. 21332411

[10] R. Sirtori , A. Pandey , A. Shukla , and C. Fallini , “A Tabletop Blast Device for the Study of the Long‐Term Consequences of Traumatic Brain Injury on Brain Organoids,” Cell Reports Methods 5 (2025): 101213, 10.1016/j.crmeth.2025.101213. 41187748 PMC12664893

[11] N. Hlavac and P. J. VandeVord , “Astrocyte Mechano‐Activation by High‐Rate Overpressure Involves Alterations in Structural and Junctional Proteins,” Frontiers in Neurology 10 (2019): 99, 10.3389/fneur.2019.00099. 30853931 PMC6395392

[12] R. Ravin , N. Y. Morgan , P. S. Blank , et al., “Response to Blast‐Like Shear Stresses Associated With Mild Blast‐Induced Brain Injury,” Biophysical Journal 117 (2019): 1167–1178, 10.1016/j.bpj.2019.07.052. 31495447 PMC6818442

[13] R. Ravin , P. S. Blank , A. Steinkamp , et al., “Shear Forces During Blast, Not Abrupt Changes in Pressure Alone, Generate Calcium Activity in Human Brain Cells,” PLoS One 7 (2012): e39421, 10.1371/journal.pone.0039421. 22768078 PMC3387147

[14] E. W. Vogel , M. B. Panzer , F. N. Morales , et al., “Direct Observation of Low Strain, High Rate Deformation of Cultured Brain Tissue During Primary Blast,” Annals of Biomedical Engineering 48 (2020): 1196–1206, 10.1007/s10439-019-02437-4. 31863230

[15] A. Vogel and V. Venugopalan , “Mechanisms of Pulsed Laser Ablation of Biological Tissues,” Chemical Reviews 103 (2003): 577–644, 10.1021/cr010379n. 12580643

[16] G. Liu , B. Li , S. Tao , et al., “Full‐Scale Explosion Experiment and Overpressure Prediction Method for 70 MPa Hydrogen Fuel Cell Vehicle Tank Under Standard Fire Test,” International Journal of Hydrogen Energy 170 (2025): 151194, 10.1016/j.ijhydene.2025.151194.

[17] K. A. Rafaels , C. R. ‘Dale’ Bass , M. B. Panzer , and R. S. Salzar , “Pulmonary Injury Risk Assessment for Long‐Duration Blasts: A Meta‐Analysis,” Journal of Trauma: Injury, Infection & Critical Care 69 (2010): 368–374, 10.1097/TA.0b013e3181e88122. 20699746

[18] R. R. Hicks , S. J. Fertig , R. E. Desrocher , W. J. Koroshetz , and J. J. Pancrazio , “Neurological Effects of Blast Injury,” Journal of Trauma 68 (2010): 1257–1263, 10.1097/TA.0b013e3181d8956d. 20453776 PMC2958428

[19] W. Bu , H. Ren , Y. Deng , et al., “Mild Traumatic Brain Injury Produces Neuron Loss That Can Be Rescued by Modulating Microglial Activation Using a CB2 Receptor Inverse Agonist,” Frontiers in Neuroscience 10 (2016): 449, 10.3389/fnins.2016.00449. 27766068 PMC5052277

[20] J. E. Burda , A. M. Bernstein , and M. V. Sofroniew , “Astrocyte Roles in Traumatic Brain Injury,” Experimental Neurology 275, no. Pt 3 (2016): 305–315, 10.1016/j.expneurol.2015.03.020. 25828533 PMC4586307

[21] D. W. Simon , M. J. McGeachy , H. Bayır , R. S. B. Clark , D. J. Loane , and P. M. Kochanek , “The Far‐Reaching Scope of Neuroinflammation After Traumatic Brain Injury,” Nature Reviews Neurology 13 (2017): 171–191, 10.1038/nrneurol.2017.13. 28186177 PMC5675525

[22] R. A. de Melo Reis , H. R. Freitas , and F. G. de Mello , “Cell Calcium Imaging as a Reliable Method to Study Neuron‐Glial Circuits,” Frontiers in Neuroscience 14 (2020): 569361, 10.3389/fnins.2020.569361. 33122991 PMC7566175

[23] M. Brini , T. Calì , D. Ottolini , and E. Carafoli , “Neuronal Calcium Signaling: Function and Dysfunction,” Cellular and Molecular Life Sciences 71 (2014): 2787–2814, 10.1007/s00018-013-1550-7. 24442513 PMC11113927

[24] A. Semyanov , C. Henneberger , and A. Agarwal , “Making Sense of Astrocytic Calcium Signals ‐ From Acquisition to Interpretation,” Nature Reviews Neuroscience 21 (2020): 551–564, 10.1038/s41583-020-0361-8. 32873937

[25] A. D. Umpierre , L. L. Bystrom , Y. Ying , Y. U. Liu , G. Worrell , and L.‐J. Wu , “Microglial Calcium Signaling Is Attuned to Neuronal Activity in Awake Mice,” eLife 9 (2020): e56502, 10.7554/eLife.56502. 32716294 PMC7402678

[26] C. Harteneck , K. Leuner , C. Harteneck , and K. Leuner , “TRP Channels in Neuronal and Glial Signal Transduction.” Neurochemistry (IntechOpen, 2014), 10.5772/58232.

[27] E. Kania , G. Roest , T. Vervliet , J. B. Parys , and G. Bultynck , “IP3 Receptor‐Mediated Calcium Signaling and Its Role in Autophagy in Cancer,” Frontiers in Oncology 7 (2017): 140, accessed September 2, 2023, https://www.frontiersin.org/articles/10.3389/fonc.2017.00140. 28725634 10.3389/fonc.2017.00140 PMC5497685

[28] A. Lilienbaum and A. Israël , “From Calcium to NF‐κB Signaling Pathways in Neurons,” Molecular and Cellular Biology 23 (2003): 2680–2698, 10.1128/MCB.23.8.2680-2698.2003. 12665571 PMC152563

[29] J.‐M. Zhang and J. An , “Cytokines, Inflammation, and Pain,” International Anesthesiology Clinics 45 (2007): 27–37, 10.1097/AIA.0b013e318034194e. 17426506 PMC2785020

[30] A. Y. Wen , K. M. Sakamoto , and L. S. Miller , “The Role of the Transcription Factor CREB in Immune Function,” Journal of Immunology 185 (2010): 6413–6419, 10.4049/jimmunol.1001829. PMC5519339 21084670

[31] J.‐Q. Kang , Z. Z. Chong , and K. Maiese , “Akt1 Protects Against Inflammatory Microglial Activation Through Maintenance of Membrane Asymmetry and Modulation of Cysteine Protease Activity,” Journal of Neuroscience Research 74 (2003): 37–51, 10.1002/jnr.10740. 13130504

[32] J.‐H. Mao , Y. Xu , B.‐W. Li , Y.‐L. Yang , Y. Peng , and F. Zhi , “Microglia Polarization in Ischemic Stroke: Complex Mechanisms and Therapeutic Interventions,” Chinese Medical Journal 134 (2021): 2415–2417, 10.1097/CM9.0000000000001711. 34669634 PMC8654435

[33] A. I. Borrachero‐Conejo , W. R. Adams , E. Saracino , et al., “Stimulation of Water and Calcium Dynamics in Astrocytes With Pulsed Infrared Light,” FASEB Journal 34 (2020): 6539–6553, 10.1096/fj.201903049R. 32202681

[34] P. Therajaran , J. A. Hamilton , T. J. O'Brien , N. C. Jones , and I. Ali , “Microglial Polarization in Posttraumatic Epilepsy: Potential Mechanism and Treatment Opportunity,” Epilepsia 61 (2020): 203–215, 10.1111/epi.16424. 31943156

[35] M. M. Maneshi , F. Sachs , and S. Z. Hua , “A Threshold Shear Force for Calcium Influx in an Astrocyte Model of Traumatic Brain Injury,” Journal of Neurotrauma 32 (2015): 1020–1029, 10.1089/neu.2014.3677. 25442327 PMC4492552

[36] N. Moro , S. Ghavim , and R. Sutton , “Massive Efflux of Adenosine Triphosphate Into the Extracellular Space Immediately After Experimental Traumatic Brain Injury,” Experimental and Therapeutic Medicine 21 (2021): 575, 10.3892/etm.2021.10007. 33850547 PMC8027727

[37] W. Bo , M. Silkunas , U. Mangalanathan , et al., “Probing Nanoelectroporation and Resealing of the Cell Membrane by the Entry of Ca2+ and Ba2+ Ions,” International Journal of Molecular Sciences 21 (2020): 3386, 10.3390/ijms21093386. 32403282 PMC7247012

[38] F. Ali and A. C. Kwan , “Interpreting In Vivo Calcium Signals From Neuronal Cell Bodies, Axons, and Dendrites: A Review,” Neurophotonics 7, no. 1 (2020): 011402, 10.1117/1.NPh.7.1.011402. 31372367 PMC6664352

[39] V. Gomez Godinez , V. Morar , C. Carmona , et al., “Laser‐Induced Shockwave (LIS) to Study Neuronal Ca2+ Responses,” Frontiers in Bioengineering and Biotechnology 9 (2021): 598896, 10.3389/fbioe.2021.598896. 33681154 PMC7928400

[40] A. P. Miller , A. S. Shah , B. V. Aperi , et al., “Effects of Blast Overpressure on Neurons and Glial Cells in Rat Organotypic Hippocampal Slice Cultures,” Frontiers in Neurology 6 (2015): 20, accessed September 2, 2023, https://www.frontiersin.org/articles/10.3389/fneur.2015.00020. 25729377 10.3389/fneur.2015.00020 PMC4325926

[41] A. P. Miller , A. S. Shah , B. V. Aperi , S. N. Kurpad , B. D. Stemper , and A. Glavaski‐Joksimovic , “Acute Death of Astrocytes in Blast‐Exposed Rat Organotypic Hippocampal Slice Cultures,” PLoS One 12 (2017): e0173167, 10.1371/journal.pone.0173167. 28264063 PMC5338800

[42] M. J. Carson , J. Cameron Thrash , and B. Walter , “The Cellular Response in Neuroinflammation: The Role of Leukocytes, Microglia and Astrocytes in Neuronal Death and Survival,” Clinical Neuroscience Research 6 (2006): 237–245, 10.1016/j.cnr.2006.09.004. 19169437 PMC2630233

[43] I. P. Karve , J. M. Taylor , and P. J. Crack , “The Contribution of Astrocytes and Microglia to Traumatic Brain Injury,” British Journal of Pharmacology 173 (2016): 692–702, 10.1111/bph.13125. 25752446 PMC4742296

[44] M. A. Gama Sosa , R. De Gasperi , G. S. Perez Garcia , et al., “Lack of Chronic Neuroinflammation in the Absence of Focal Hemorrhage in a Rat Model of Low‐Energy Blast‐Induced TBI,” Acta Neuropathologica Communications 5 (2017): 80, 10.1186/s40478-017-0483-z. 29126430 PMC6389215

[45] S. A. Bhat , R. J. Henry , A. C. Blanchard , B. A. Stoica , D. J. Loane , and A. I. Faden , “Enhanced Akt/GSK‐3β/CREB Signaling Mediates the Anti‐Inflammatory Actions of mGluR5 Positive Allosteric Modulators in Microglia and Following Traumatic Brain Injury in Male Mice,” Journal of Neurochemistry 156 (2021): 225–248, 10.1111/jnc.14954. 31926033 PMC7386074

[46] Z. V. Zheng , J. Chen , H. Lyu , et al., “Novel Role of STAT3 in Microglia‐Dependent Neuroinflammation After Experimental Subarachnoid Haemorrhage,” Stroke and Vascular Neurology 7 (2022): 62–70, 10.1136/svn-2021-001028. 34645687 PMC8899684

[47] M. D. LeComte , I. S. Shimada , C. Sherwin , and J. L. Spees , “Notch1‐STAT3‐ETBR Signaling Axis Controls Reactive Astrocyte Proliferation After Brain Injury,” Proceedings of the National Academy of Sciences 112 (2015): 8726–8731, 10.1073/pnas.1501029112. PMC4507218 26124113

[48] R. A. Chiareli , G. A. Carvalho , B. L. Marques , et al., “The Role of Astrocytes in the Neurorepair Process,” Frontiers in Cell and Developmental Biology 9 (2021): 665795, 10.3389/fcell.2021.665795. 34113618 PMC8186445

[49] A. Misistia , M. Skotak , A. Cardenas , E. Alay , N. Chandra , and G. H. Kamimori , “Sensor Orientation and Other Factors Which Increase the Blast Overpressure Reporting Errors,” PLoS One 15 (2020): e0240262, 10.1371/journal.pone.0240262. 33031423 PMC7544144

[50] I. Cernak , “Blast Injuries and Blast‐Induced Neurotrauma: Overview of Pathophysiology and Experimental Knowledge Models and Findings.” in Brain Neurotrauma: Molecular, Neuropsychological, and Rehabilitation Aspects, eds. F. H. Kobeissy (CRC Press/Taylor & Francis, 2015), http://www.ncbi.nlm.nih.gov/books/NBK299193/. 26269895

[51] M. Fricker , A. M. Tolkovsky , V. Borutaite , M. Coleman , and G. C. Brown , “Neuronal Cell Death,” Physiological Reviews 98 (2018): 813–880, 10.1152/physrev.00011.2017. 29488822 PMC5966715

[52] M. M. Rahman , M. R. Islam , M. Yamin , et al., “Emerging Role of Neuron‐Glia in Neurological Disorders: At a Glance,” Oxidative Medicine and Cellular Longevity 2022 (2022): 3201644, 10.1155/2022/3201644. 36046684 PMC9423989

